# Bronchobiliary fistula following radiofrequency ablation for liver metastases from breast cancer: A case report and literature review

**DOI:** 10.1097/MD.0000000000012760

**Published:** 2018-10-26

**Authors:** Xue-Jiao Xi, Yi Zhang, Yun-Hong Yin, Hao Li, De-Dong Ma, Yi-Qing Qu

**Affiliations:** Department of Respiratory Medicine, Qilu Hospital of Shandong University, Jinan, Shandong Province, China.

**Keywords:** bilioptysis, breast cancer, bronchobiliary fistula, liver metastases, radiofrequency ablation

## Abstract

**Rationale:**

Bronchobiliary fistula (BBF) is a rare clinical condition which is characterized by a channel between biliary tract and bronchial tree. BBF can present with fever, dyspnea, and cough. However, it can be easily misdiagnosed as biliary vomiting, dyspnea, or even severe pneumonia.

**Patient concerns:**

A 53-year-old woman was diagnosed with breast cancer in April 2011 and underwent radical mastectomy and lymph node dissection, chemotherapy, and radiotherapy. Unfortunately, the patient suffered from bone metastasis during the 1st year and liver metastasis during the 2nd year after radical mastectomy. In 2013, the patient underwent transcatheter arterial chemoembolization therapy twice for liver metastasis. The patient was then treated with radiofrequency ablation (RFA) in 2016. Unfortunately, the patient developed a cough with bitter-tasting yellow sputum and chest tightness 2 weeks after the RFA treatment. Approximately 6 months later, the patient still complained of a cough with yellow sputum and persistent chest tightness. The patient was then admitted to our department.

**Diagnoses:**

The presence of bile in the sputum supported a diagnosis of BBF. Bronchoscopy was performed, and the presence of bile in the lavage fluid confirmed the diagnosis of BBF.

**Interventions:**

The patient was treated with antibiotics including sulbactam, cefoperazone, levofloxacin and meropenem, was well as hepatoprotectants, nutritional support and other supportive treatments in our department.

**Outcomes:**

The patient died because of liver failure.

**Lessons:**

This case demonstrates that we should consider the possibility of BBF when patients experience a recurrent cough with discolored sputum after RFA. In particular, a diagnosis of BBF should be considered in patients who do not respond to antibiotic treatment.

## 1 Introduction

Bronchobiliary fistula (BBF) is a rare clinical condition that is defined as a channel between the biliary tract and the bronchial tree. BBF can be caused by congenital bronchobiliary diseases, hepatic hydatid disease, gallstones or tumors, or can occur for iatrogenic reasons such as liver resection and radiofrequency ablation (RFA).^[1]^ BBF was first reported in 2002, and only 1 in 3670 patients with hepatic tumors suffered from BBF after RFA.^[2]^ A review of the literature from 2002 to date showed that a total of 13 patients with BBF after RFA have been reported (Table 1). Clinically, BBF usually presents with fever, dyspnea, coughing, and bile in the sputum (bilioptysis). Of these symptoms, bilioptysis is the most common symptom.^[3]^ Due to the low incidence rate and the lack of understanding of this disease, BBF can easily be misdiagnosed as biliary vomiting, dyspnea, or severe pneumonia. The treatment for BBF is also difficult and is usually associated with high mortality. RFA is one of the most widely used minimally invasive surgical technique for small hepatocellular carcinoma tumors (<3 cm in diameter), and RFA works by heating the local tumor tissue to cause coagulation and necrosis that ultimately kill the tumor cells.^[4]^ Due to the widespread use of RFA, reports of BBF cases have been increasing in recent years. Here, we present a case of BBF following RFA treatment in a patient with liver metastases from breast cancer and summarize the common clinical characteristics, diagnostic criteria, and treatment strategies for BBF.

**Table 1:**
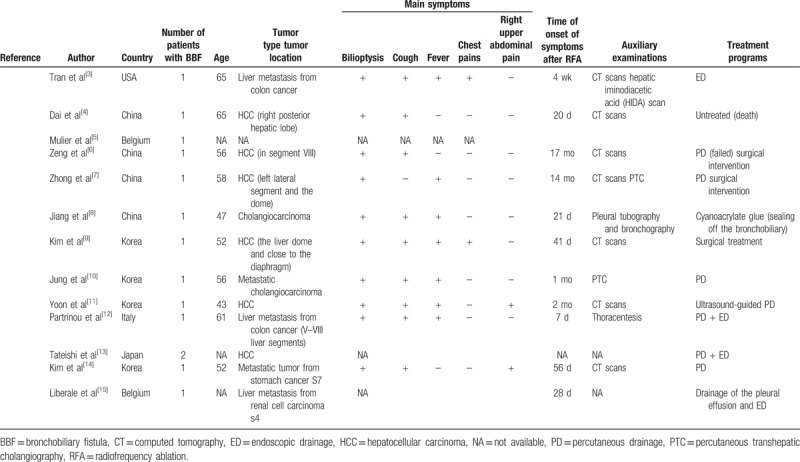
Summary of cases of bronchobiliary fistula after radiofrequency ablation of liver tumors.

## 2 Case report

A 53-year-old woman was diagnosed with left breast cancer in April 2011, and radical mastectomy and lymph node dissection were performed, followed by radiotherapy and chemotherapy. In May 2012, a chest CT and radionuclide bone scan demonstrated the presence of bone metastases (L3 lumbar vertebrae). In March 2013, liver metastases were found, and the patient underwent transcatheter arterial chemoembolization twice. In May 2016, the patient was treated with RFA for the metastatic liver lesions. Unfortunately, 2 weeks later, the patient suffered from a cough with yellow, sticky, bitter-tasting sputum, chest tightness, shortness of breath, and worsening symptoms after exercise. The patient was admitted to a local hospital and was diagnosed with a mycotic and bacterial pulmonary infection. The patient underwent treatment with imipenem (1 g, intravenous drip every 12 hours) for 10 days and empirical voriconazole (200 mg, intravenous drip every 12 hours) for 15 days. However, after the treatment with imipenem and empirical voriconazole, the patient developed a fever with a temperature of 42°C without associated shivering. The fever was alleviated with an intravenous injection of dexamethasone (5 mg). However, the intermittent fever lasted for 40 days, and the patient's body temperature ranged between 36°C and 38°C. During this period, the patient received antifungal treatment (oral voriconazole 200 mg twice a day). After September 2016, the patient had no fever, but had a persistent cough with yellow sputum, and wheezing after exercise. The patient was then admitted to our department in November 2016. The patient had an obvious cough with yellow-green sputum, and mild abdominal discomfort, but no symptoms of fever, hemoptysis, nausea, vomiting, or jaundice.

On physical examination, the left breast was absent, and an annular scar of approximately 7 cm was present. Breath sounds were decreased in the right lower lung field. The abdomen was soft, but the upper abdomen was tender and had rebound pain. The patient had a positive Murphy sign, hepatomegaly <4 cm under the rib arch, and edema of both lower extremities. The remainder of the physical examination was normal. The patient's laboratory tests showed a white blood cell count of 9.72 × 10^9^/L (normal range: 3.5–9.5 × 10^9^/L), a neutrophil count of 7.77 × 10^9^/L (normal range: 1.8–6.3 × 10^9^/L), a lymphocyte count of 0.98 × 10^9^/L (normal range: 1.1–3.2 × 10^9^/L), a monocyte count of 0.92 × 10^9^/L (normal range: 0.1–0.6 × 10^9^/L), and an erythrocyte sedimentation rate of 10 mm/h (normal range: 0–18 mm/h). Liver function testing revealed an aspartate transaminase of 93 IU/L (normal range: 13–35 IU/L), an alanine aminotransferase of 23 IU/L (normal range: 7–40 IU/L), an alkaline phosphatase of 601 IU/L (normal range: 50–135 IU/L), an albumin of 30 g/L (normal: 40.0–55.0 g/L), a total bilirubin of 21.9 μmol/L (normal range: 5–21 μmol/L), a direct bilirubin of 13.3 μmol/L (normal range: <6 μmol/L), and an indirect bilirubin of 8.6 μmol/L (normal range: 2–15 μmol/L). Coagulation testing showed a prothrombin time of 18.3 seconds (normal range: 8.0–13.8 seconds), a prothrombin time activity of 48% (normal range: 70–140%), and a fibrinogen of 1.82 g/L (normal range: 2.00–4.00 g/L). Values for tumor-associated markers included the following: 165.80 ng/mL (normal range: 0.1–3.3 ng/mL) for nonsmall-cell lung cancer associated antigen, 5.24 ng/mL (normal range: 0–20 ng/mL) for alpha-fetoprotein, >1000 μg/L (normal range: 0–5 μg/L) for carcinoembryonic antigen, >300 U/mL (normal: 0–25 U/mL) for carbohydrate antigen (CA-153), and 299.10 U/mL (normal range: 0–35 U/mL) for carbohydrate antigen (CA-125). A fungal d-glucan test was negative, and serologic examination for aspergillus was negative (Table 2). There was no fungal or bacterial growth on culture of the sputum. Notably, the total bilirubin level of the sputum was 120 μmol/L, and direct bilirubin was 80 μmol/L.

**Table 2:**
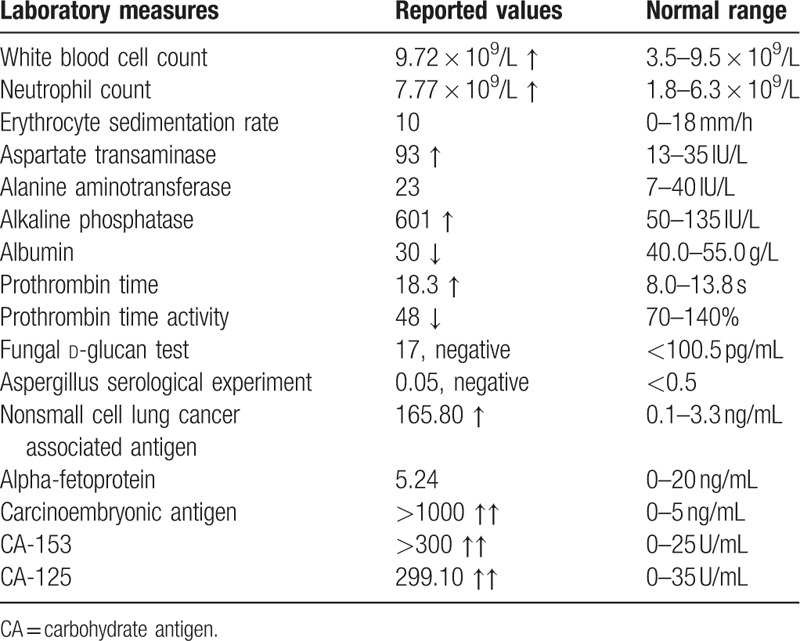
Laboratory tests at admission.

**Figure 1:**
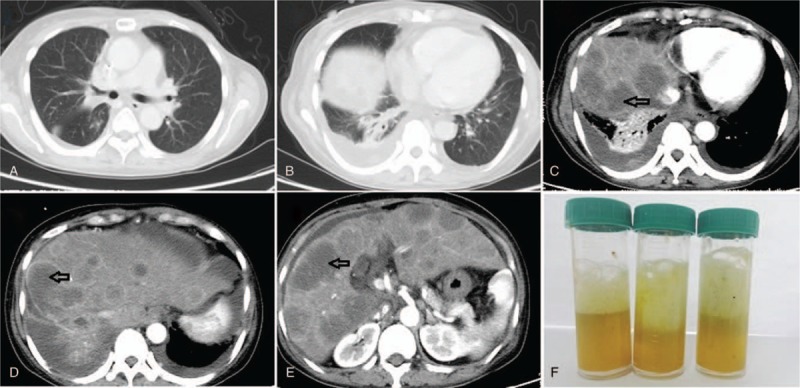
(A, B) High-resolution computed tomography images of the chest and abdomen showing lower right lung inflammation, bilateral pleural effusion, and atelectasis of part of the right lung. (C–E) High-resolution computed tomography images of the chest and abdomen showing multiple intrahepatic metastases, particularly near the right hepatic dome. (F) Bronchoscopy results showing yellow airway yellow secretions. Bronchoscopic lavage specimens were collected, and the lavage fluid was positive for bile.

High-resolution computed tomography (HRCT) of the chest and abdomen showed enlargement of the right lung hilum and mediastinum with slightly enlarged lymph nodes, pleural thickening of both lungs, multiple nodular enhancements in the right pleura, bilateral pleural effusion, atelectasis of part of the right lung, ascites, and multiple intrahepatic metastases (Fig. 1A–E). Bronchoscopy showed the presence of yellow airway secretions, tracheal mucosal hypertrophy of the basal segment of the right lower lobe, obvious swelling, and no organisms. There were no abnormalities in the visible portion of the remaining bronchus. Furthermore, lavage specimens were collected, and the lavage fluid was positive for bile (Fig. 1F).

The presence of bile in the sputum and bronchial lavage fluid strongly supported the diagnosis of BBF.^[5]^ Therefore, we recommended that the patient undergo laparotomy to confirm the diagnosis of BBF and to allow for therapeutic intervention during laparotomy. Unfortunately, after clinical evaluation, the multidisciplinary team thought that the patient could not tolerate a laparotomy. They suggested that endoscopic nasobiliary drainage (ENBD) might provide stronger evidence for further confirmation of the diagnosis. However, the patient's family elected to forego further examination and treatment after learning of the condition of the patient.

During this period of hospitalization, the patient was first treated with antibiotics including sulbactam and cefoperazone (2 g/12 h) and levofloxacin (0.6 g/d) for 5 days. In addition, her antibiotic regimen was then changed to meropenem for 4 days. She was also prescribed hepatoprotectants, nutritional support and other supportive treatments. After treatment for 10 days, the symptoms of cough with yellow-green sputum and mild abdominal discomfort improved. On the 10th day of hospitalization, she was discharged home. We recommended that the patient should continue receive treatment for her primary disease after discharge. In addition, we also recommended that the patient should receive future treatment with laparotomy once her general condition improved. The patient was followed up for 6 months. Unfortunately, the patient died because of liver failure.

## 3 Discussion

**Figure 2:**
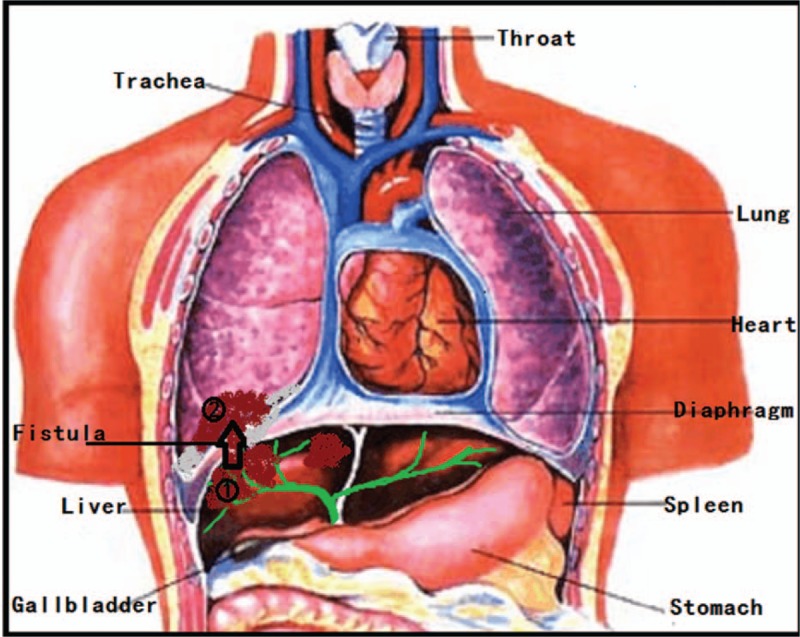
Diagram of bronchobiliary fistula after radiofrequency ablation in a patient with liver cancer. When the ablation lesion is close to the top of the diaphragm, serious lesions such as phrenic and liver abscesses, ulcers, and necrosis can easily occur. In addition, inflammatory adhesions and necrosis of the bile duct, diaphragm and surface of the lung can lead to the development of a pulmonary abscess. Such an abscess can rupture in the bronchus and eventually form a biliary fistula between the liver and the bile duct. (This figure was revised from a version from www.jiyiphoto.net.)

The RFA, which is as a minimally invasive treatment, is relatively safe, and BBF is a rare complication of RFA in the clinic. Due to the wide application of RFA, the number of cases of BBF has increased in recent years. In general, RFA usually causes the ablated area to become completely necrotic and liquefied and when the lesion is close to the top of the diaphragm, lesions such as phrenic and liver abscesses, ulcers, and necrosis can easily form.^[16]^ In addition, inflammatory adhesions and necrosis of the bile duct, diaphragm and surface of the lung can lead to the development of pulmonary abscesses that collapse in the bronchus and eventually form a biliary fistula between the liver and bile duct.^[17]^ The site of BBF is often located on the right side for the following reasons: The right hepatic duct is more easily visible than the left. The vertical portion of the duct (particularly for the right posterior superior bile duct) is close to the diaphragm. The upper and lower portions of the right liver often have adhesions to the top of the diaphragm, resulting in closure of the top of the diaphragm. There is no room for movement, so abscesses can perforate (Fig. 2).

A review of the literature from 2002 to date showed that a total of 13 patients with BBF after RFA have been reported (Table 1). The average age of patients with BBF after RFA was 55 years (range 43–65 years). The time of BBF occurrence after RFA ranged from 4 days to 17 months. The formation of biliary neoplasia, biliary tract infections, and biliary obstructions were all risk factors that affected the timing of onset of symptoms. The clinical symptoms of BBF included bilioptysis (10/10), cough (9/10), fever (7/10), chest pain (2/10), and right upper abdominal pain (2/10). Because of the low incidence rate, BBFs are often overlooked and misdiagnosed in the early stages of the disease. BBF is easily misdiagnosed as biliary vomiting, dyspnea, or severe pneumonia.^[16]^ The presence of bile in the sputum can be found on biochemical examination of the sputum. As described in the present case, our patient had a cough with yellow sputum, fever, chest tightness, right upper abdominal pain, and a poor response to antibiotic treatment. In combination with the patient's medical history of RFA, we considered the possibility of BBF and cholestatic pneumonia. Ultimately, the presence of bile in the sputum and bronchial lavage fluid strongly supported the diagnosis of BBF.

To further clarify the diagnosis, it was necessary to perform a complete imaging examination. Chest radiographs often show abnormal manifestations, such as pleural effusion, atelectasis of the right basal lung, or presence of pulmonary abscesses.^[11]^ CT scans may not show the fistula directly but may reveal indirect manifestations such as pneumonia, pleural effusion, liver abscesses, and bile duct stones. CT scans can also show hepatic abscesses, abnormal bile flow, and gas–fluid interfaces, which are highly suggestive of BBF.^[3]^ This patient underwent HRCT of the chest and abdomen. The patient's CT images showed lower right lung inflammation, bilateral pleural effusion, atelectasis of part of the right lung, and multiple intrahepatic metastases, particularly near the right hepatic dome (Fig. 1A–D). A large tumor close to the hepatic dome has been found to be a predisposing factor for BBF following RFA.^[18]^ In the past, the patient underwent multiple interventional procedures and had prolonged hypoproteinemia, and the thermal effect of RFA promoted the occurrence of BBF. The presence of a fistula is rarely detected by bronchoscopy. Although bronchoscopy is not routinely performed, it has certain diagnostic indications. Bronchoscopy revealed greenish bile in the bronchus, and the bronchial lavage specimen was bilious, which together made the diagnosis of BBF more definitive.^[19]^ Cases have been reported in which no bile was present in the lavage fluid, but a diagnosis of biliary pneumonia and an indirect diagnosis of BBF were made based on transbronchial biopsy findings of brown bilious crystals surrounded by neutrophils.^[20]^ Diagnostic bronchoscopy was performed for our patient, and the bronchial lavage fluid was positive for bile; thus, the diagnosis of BBF was confirmed (Fig. 1F).

Localization of BBFs can be achieved by using magnetic resonance cholangiopancreatography (MRCP), percutaneous transhepatic cholangiography (PTC), endoscopic retrograde cholangiography (ERCP), or technicium-99m hepatobiliary iminodiacetic acid (^99m^Tc-HIDA) cholescintigraphy.^[10]^ MRCP is a safe and noninvasive diagnostic technique for visualizing the biliary tract, but it cannot be used in the treatment of biliary diseases.^[21]^ PTC is a more invasive examination that can more fully and intuitively demonstrate the existence of BBF, and it can evaluate bile drainage at the same time.^[22]^ ERCP is used to visualize the contrast agent flowing through the diaphragm into the chest and bronchus to diagnose BBF, and biliary obstructions can be displayed by using endoscopic retrograde biliary drainage (ERBD).^[23]^^99m^Tc-HIDA cholescintigraphy provides information on the anatomic development and function of the biliary tract and can assess the integrity of the biliary tract. It is rarely used because of its high cost.^[24]^ Considering the general condition of our patient, the multidisciplinary team thought such a test would not be tolerated, and the risk was too high. Therefore, we decided perform any further diagnostics after communicating with her family members.

Current trends in the treatment of BBF are changing from traditional surgery to minimally invasive therapies that have been proven to be effective and safe. Minimally invasive treatments usually include ERBD, ENBD, sphincterotomy, and percutaneous drainage (PD).^[6,25]^ Most patients with BBF can experience relief of their symptoms after biliary drainage. In 2002, Tran et al first reported endoscopic sphincterotomy and stent placement that led to a significant improvement in symptoms in the treatment of a bronchobiliary fistula secondary to RFA.^[3]^ Jung et al reported that a patient with BBF complicated by an abscess after RFA who was cured by draining a liver abscess and percutaneous transhepatic biliary drainage. However, open surgery should be the first choice when minimally invasive therapy has failed.^[6,7]^ ENBD is a technique that uses endoscopic insertion of a nasobiliary tube to drain bile so that the fistula is successfully closed in a short time by reducing the pressure of the bile.^[12]^ Open surgery is often required if the drainage treatments fail. Surgical methods include drainage of subphrenic abscesses or liver abscesses, resection of the fistula, and repair of the fistula and empyema. A few patients need pulmonary segmental lobectomy or pulmonary lobectomy.^[6]^ Direct bronchoscopy has been reported to be a new treatment method, but more clinical experience is still necessary.^[25]^ In this case, the multiple disciplinary team suggested that minimally invasive treatment might be used as palliative treatment due to patient's the poor clinical condition with advanced malignant tumors, poor general condition, and inability to undergo surgery. ENBD might provide stronger evidence for further confirmation of the diagnosis of BBF and potential treatment. However, the patient's family decided to forego further examinations and treatment after learning of the condition of the patient.

## 4 Conclusion

Here we report a rare case of BBF after RFA in a patient who was diagnosed with liver metastasis from breast cancer. Because BBF after RFA is very rare in clinical setup, it was initially misdiagnosed as pneumonia and the treatment was delayed. This was a warning to us that we should consider the possibility of BBF when patients experience a recurrent cough with discolored sputum after RFA. In particular, a diagnosis of BBF should be considered in patients who do not respond to antibiotic treatment. The timely examination of bilioptysis is beneficial to the diagnosis.

## Author contributions

**Resources:** Yi Zhang.

**Supervision:** Yi-Qing Qu.

**Writing – original draft:** Xue-Jiao Xi.

**Writing – review & editing:** Yi Zhang, Yun-Hong Yin, Hao Li, De-Dong Ma.

Yi-Qing Qu orcid: 0000-0002-6297-3989.
